# Assessing the cost-effectiveness of HPV vaccination strategies for adolescent girls and boys in the UK

**DOI:** 10.1186/s12879-019-4108-y

**Published:** 2019-06-24

**Authors:** Samik Datta, Joshua Pink, Graham F. Medley, Stavros Petrou, Sophie Staniszewska, Martin Underwood, Pam Sonnenberg, Matt J. Keeling

**Affiliations:** 1 Zeeman Institute: SBIDER, Warwick Mathematics Institute and School of Life Sciences, The University of Warwick, Coventry, CV4 8UW UK; 2 National Institute of Water and Atmospheric Research, Wellington, 6021 New Zealand; 3 Warwick Clinical Trials Unit, Warwick Medical School, The University of Warwick, Coventry, CV4 8UW UK; 4 Department for Global Health and Development, London School of Hygiene and Tropical Medicine, London, WC1H 9SH UK; 5 Royal College of Nursing Research Institute, Warwick Medical School, The University of Warwick, Coventry, CV4 8UW UK; 6 Research Department of Infection and Population Health, University College London, London, WC1E 6JB UK

**Keywords:** HPV, Sexually transmitted infection, Human papillomavirus, Epidemiology, Modelling, MCMC, cost-effectiveness, Vaccination

## Abstract

**Background:**

Human papillomavirus (HPV) is the most widespread sexually transmitted infection worldwide. It causes several health consequences, in particular accounting for the majority of cervical cancer cases in women. In the United Kingdom, a vaccination campaign targeting 12-year-old girls started in 2008; this campaign has been successful, with high uptake and reduced HPV prevalence observed in vaccinated cohorts. Recently, attention has focused on vaccinating both sexes, due to HPV-related diseases in males (particularly for high-risk men who have sex with men) and an equity argument over equalising levels of protection.

**Methods:**

We constructed an epidemiological model for HPV transmission in the UK, accounting for nine of the most common HPV strains. We complemented this with an economic model to determine the likely health outcomes (healthcare costs and quality-adjusted life years) for individuals from the epidemiological model. We then tested vaccination with the three HPV vaccines currently available, vaccinating either girls alone or both sexes. For each strategy we calculated the threshold price per vaccine dose, i.e. the maximum amount paid for the added health benefits of vaccination to be worth the cost of each vaccine dose. We calculated results at 3.5% discounting, and also 1.5%, to consider the long-term health effects of HPV infection.

**Results:**

At 3.5% discounting, continuing to vaccinate girls remains highly cost-effective compared to halting vaccination, with threshold dose prices of £56-£108. Vaccination of girls and boys is less cost-effective (£25-£53). Compared to vaccinating girls only, adding boys to the programme is not cost-effective, with negative threshold prices (-£6 to -£3) due to the costs of administration. All threshold prices increase when using 1.5% discounting, and adding boys becomes cost-effective (£36-£47). These results are contingent on the UK’s high vaccine uptake; for lower uptake rates, adding boys (at the same uptake rate) becomes more cost effective.

**Conclusions:**

Vaccinating girls is extremely cost-effective compared with no vaccination, vaccinating both sexes is less so. Adding boys to an already successful girls-only programme has a low cost-effectiveness, as males have high protection through herd immunity. If future health effects are weighted more heavily, threshold prices increase and vaccination becomes cost-effective.

**Electronic supplementary material:**

The online version of this article (10.1186/s12879-019-4108-y) contains supplementary material, which is available to authorized users.

## Background

Human papillomavirus (HPV) is the world’s most common sexually transmitted infection, with the majority of people being infected at some point in their lifetime [1, 2]. At any time, up to 44% of sexually active women are infected [3, 4]. Although for most people it is symptomless and they recover from the infection with no adverse effects, for a small proportion the infection may lead to adverse health sequelae. Of the 200 strains known and 120 catalogued thus far [5, 6], at least 18 have been labelled ‘high-risk’ HPV types due to their associations with development of different cancers [7]; in particular, strains 16 and 18 are associated with the majority of cervical cancers [8, 9]. The high health and economic burden caused by HPV infection has led to many countries initiating HPV vaccinations campaigns.. Available vaccines include: a bivalent vaccine protecting against HPV-16 and HPV-18 (Cervarix^®^) and a quadrivalent vaccine, which also protects against HPV-6 and HPV-11 (Gardasil^®^), both of which are linked to genital warts in both sexes [10]. A nonavalent vaccine (Gardasil 9^®^) has recently been approved for administration which, in addition to the aforementioned types, also protects against HPV types 31, 33, 45, 52, and 58. Uptake in different countries has varied considerably since the introduction of HPV vaccination. The United Kingdom (UK), which is the primary focus of this work, has relatively high national uptake rates of around 76-90% [11]. Australia and Portugal also have relatively high uptake rates (70-80%), while some countries such as the USA, France and New Zealand have low uptake (21-47%) [12–15]. Some low and middle income countries such as Rwanda and Bhutan have also successfully set up programmes with extremely high reported uptake rates of 93-99% [16, 17].

Vaccinating when young, before sexual debut, is optimal [18, 19]. Early immunisation programmes targeted young teenage girls, with the focus on reducing incidence of cervical cancer later in life. In 2007, Australia targeted a wider age range of girls / women aged 12-26 as part of a limited catch-up programmes [20], and improvements in overall health outcomes have been shown in boys as well as girls due to indirect protection [21]. In the UK, teenage girls (aged 12-13) have been vaccinated in school since 2008, initially using the bivalent vaccine, replaced by the quadrivalent since 2011 [22]. National vaccination coverage has been consistently high since vaccination began, in the region of 76-90% [11, 23], making the programme one of the most successful globally in combating HPV. A catch-up programme for older girls was also implemented for three years (2008-2011), whereby 13-18 year old girls who missed out due to their age were also offered the vaccine [24]. Original models [19, 25] predicted it would be cost-effective to vaccinate teenage girls, provided the duration of protection was at least ten years, as it would lower the incidence of subsequent health conditions arising from HPV infection, in particular cervical cancer, which is a major health burden [26]. Recently, the decision was made to reduce the three-dose schedule (two primers plus one booster) to two doses (one primer plus one booster), following immunological evidence for comparable efficacy [27, 28] and improved cost-effectiveness [29].

In 2011 Giuliano et al. [30] questioned whether or not it would be cost-effective to include additional target groups to vaccination programmes, given that the HPV vaccine is efficacious in both sexes. A 2016 meta-analysis by Brisson et al. [31] provides an overview of the herd effects of vaccinating girls. Studies have shown some groups to have relatively high prevalence of HPV and significant health effects, including HIV-positive individuals [32, 33], and men who have sex with men (MSM) [34, 35]; the latter benefit less from the herd immunity generated by vaccinating girls than heterosexual men. Modelling has shown (as is intuitive) that increasing targets for vaccination, such as adding boys to a girls-only programme [18], and including adults [36], leads to further reductions in prevalence. However, in terms of cost-effectiveness, results of health economic analyses of contrasting programmes have generated less clear cut results. Most studies show vaccination of girls is highly cost-effective in many countries despite different cost-effectiveness thresholds for health improvements (e.g.[19, 37, 38], while the majority of studies that have proposed adding males to a female-only vaccination programme found it was less cost-effective compared to vaccinating only females [39, 40] and, in most cases, not cost-effective using standard thresholds of willingness to pay [24, 41–43]. A US study found that it would only be cost-effective if coverage in girls was around 20% [44]. Research in Australia also showed a limited impact of boys’ vaccination on HPV infections and related cancers in males [45], although Australia subsequently became the first country to adopt national gender-neutral vaccination of boys as well as girls [46]; New Zealand also added boys to the national programme in 2016. An analysis of vaccinating MSM in the USA found that it could be cost-effective, although it assumed the current vaccination programme in girls had no effect on HPV prevalence in MSM [47]. There thus remain many questions as to the cost-effectiveness of expanding the current girls-only strategy in the UK; we focus on the specific problem of adding teenage boys to the current vaccination programme.

Here we present an analysis of HPV infection and vaccination, to estimate the incremental cost-effectiveness of vaccinating boys as well as girls. The study consists of three parts: firstly, the fitting of parameters associated with HPV transmission, infection and recovery, by use of an epidemiological model incorporating sexual partnerships between individuals, matched to multiple HPV prevalence data sources. Secondly, the simulation of a range of vaccination strategies using the parameters from the above model. Thirdly, an economic analysis of the different strategies, taking into account the potential consequences (including health-related quality of life and cost implications) of HPV infections, to assess the cost-effectiveness of each vaccination strategy. We have not included potential changes to the UK’s cervical cancer screening service that might be precipitated by any future reduction in HPV prevalence. In this regard we follow the earlier analysis of female-only vaccination [19] and focus on the epidemiological and economic impacts of vaccination.

## Methods

### Data

#### HPV prevalence data pre-vaccination

To fit the transmission model to HPV prevalence rates before vaccination programmes were introduced, a variety of data, across a range of countries, was used. In total, results from 13 detailed epidemiological studies were used; information is given in Additional file 3: Table S1. Data consisted of either prevalence of serum antibodies against HPV types, or HPV DNA presence in the epithelial layer. The sex and age (groups) of individuals in each study were generally known, as well as, in the case of the studies by Nielson et al. [48] and Tanton et al. [49], sexual activity of individuals, and in the case of the study by King et al. [35], sexual orientation. These data were used to infer the infection parameters of our model including type-specific transmission probabilities.

#### Partnership rates

To model partnership behaviour of individuals, we used data from National Survey of Sexual Attitudes and Lifestyles (NATSAL) 2 and 3 [50]: UK-wide surveys of sexual behaviour. We fitted distributions to stratified data which allowed us to determine the annual rates of forming new sexual partners where there is unprotected intercourse, as well as the likely ages of these partners. Individuals were stratified by age, sex, sexual preference and previous sexual experience. For all simulations, we used NATSAL-2 distributions up to 2010, and NATSAL-3 distributions simulating forwards from 2010.

### Epidemiological model

In the following section we describe the epidemiological model in brief; the transmission framework is explored in more detail in Datta et al. [50], and in Additional file 1: Appendix S1.

We used an individual-based modelling framework, with SIRS-V (Susceptible - Infected - Recovered - Susceptible - Vaccinated) dynamics, thus accounting for both short-duration natural immunity and longer-lasting protection due to vaccination. Populations of 50,000 individuals were generally modelled; this population size was a compromise between stochastic uncertainty and speed of the computationally intensive simulations. We used yearly data from Natsal-2 and Natsal-3 to determine distributions for the rates of new partnerships that involve unprotected sex and could therefore allow the spread of HPV [50]. Different distributions were defined depending on four personal characteristics: age (years), sex (male/female), sexual orientation (heterosexual/any other) and previous sexual experience (yes/no). Individuals in the population were given a risk-percentile, which determined the values extracted from the distribution of rates, with the distribution defined by the individual’s characteristics (e.g. age, sex, etc). This meant that high risk-percentile individuals consistently had high rates of new partnerships relative to their peers. However, we know that individual behaviour patterns can change (e.g. by starting a long-term relationship); we therefore allowed risk-percentiles to be randomly redrawn with a low age- and sex-dependent probability - this allowed us to capture longer term (5-year and lifetime) behaviour from the NATSAL surveys.

The model was generally run for 100 years, allowing individuals to age, form new partnerships, become infected and recover. When an individual stochastically picked a new partner, the characteristics of the new partner were probabilistically determined by the status of the individual choosing. If the chosen partner was infected with one or more types of HPV, these could be stochastically transferred, with separate transmission probabilities for each type and with asymmetric transmission between the sexes [51]. Once infected, an individual had a rate of recovery, equal for all types, and may have generated a detectable serological response (separate probabilities for males and females) allowing us to match the model to serological data. After recovery, there was a period of strain-specific natural immunity before the individual became susceptible once more. In total, the model required fifteen HPV-specific parameters to be inferred from the empirical data.

We used 13 datasets to fit the parameters in the epidemiological model; the datasets used are listed in Additional file 3: Table S1. All data were either serological data (detecting the presence / absence of antibodies which the individual produced naturally after a previous or current infection), or DNA data (indicative of a current HPV infection). Although serological data were more widely available, the probability of producing antibodies following an infection is not high; probabilities are thought to be around 60% for females [52] and 30% for males, although some studies report much lower type-specific rates[53]. These serological probabilities were inferred as part of the model-fitting process. DNA data, on the other hand, was considered more reliable, and we therefore assumed 100% sensitivity and specificity of these data. The model parameters were inferred using a Bayesian Markov Chain Monte Carlo (MCMC) framework and a standard Metropolis-Hastings algorithm. Likelihoods were generated by assuming that the data reflected a binomial sample of the model population.

#### Simulating vaccination strategies

For predicting the impact of vaccination, we followed the UK’s Joint Committee on Vaccination and Immunisation (JCVI) guidelines and used the ‘best’ parameters from the model fitting (i.e. the mode from the posterior for each fitted parameter). We then estimated future levels of HPV in the population for different vaccination strategies. Although the parameters used for each run were identical, due to the stochastic nature of the simulation, there was considerable variability between runs necessitating multiple simulations (500 runs per vaccination strategy). When simulating future vaccination scenarios, we used available uptake rates to simulate the girls-only vaccination that had occurred in the period 2008-2016 inclusive, using the bivalent vaccine until 2012, and quadrivalent after that [11, 54]; we also took into account the catch-up campaigns targeting girls aged 13-18 that occurred in 2008-11. (Uptake rates for both the routine and catch-up campaigns are shown in Additional file 4: Table S2.) For both campaigns, we conservatively assumed that only girls who received all three HPV doses were protected. The three available vaccines, are each assumed to confer complete protection for their target types (16 and 18 for the bivalent vaccine; 6, 11, 16 and 18 for the quadrivalent vaccine; and 6, 11, 16, 18, 31, 33, 45, 52 and 58 for the nonavalent vaccine) but differing levels of cross-protection to any remaining modelled HPV types (Additional file 5: Table S3). Forward simulations generated lifetime histories of individuals, for use in the economic analysis (see next section). Note that we assumed a two dose schedule (vaccine plus booster) for all future strategies, following evidence that this is likely to be more cost-effective than three doses if vaccine protection is at least 20 years [29]. We assumed for simplicity that both doses were given simultaneously to individuals, and protection began immediately. Therefore we did not have to explicitly model the first dose.

The following vaccination strategies were simulated into the future, using the bivalent, quadrivalent or nonavalent vaccine. We define ‘historical vaccination’ as simulating girls-only vaccination for 2008-2016, with uptake rates for the main and catch-up programmes taken from UK data (Additional file 4: Table S2), and the respective new strategies began at the start of 2017:

1. Halted vaccination: historical vaccination, followed by a halting of all vaccination in 2017;
2. Girls: historical vaccination, followed in 2017 by selecting 85% of 12-year-old females to be vaccinated at the start of each year (based on predictions from JCVI on future uptake rates);
3. Girls and boys: historical vaccination, followed in 2017 by selecting 85% of 12-year old girls and 85% of 12-year old boys to be vaccinated at the start of each year (this assumed that boys’ uptake would be equal to that of girls);
4. Girls and boys equal: historical vaccination, followed in 2017 by selecting 42.5% of 12-year old girls and 42.5% of 12-year old boys at the start of each year to be vaccinated (hence an equal level of vaccination as girls’ vaccination (strategy 2));
5. Girls naïve: no historical vaccination, and vaccinating 60% of 12-year old girls from 2008 onwards;
6. Girls and boys naïve: no historical vaccination, and vaccinating 60% of 12-year old girls and 60% of 12-year old boys from 2008 onwards.

Halted vaccination was included, not as a plausible future strategy, but so that the threshold vaccine prices could be compared to a baseline (analogous to starting a new vaccination programme compared to not beginning one). The final three scenarios were designed to provide a scientific understanding of the generic conditions under which a gender-neutral vaccination programme would be cost-effective. Strategy 4 represents countries (like the UK) that have already commenced a girls-only vaccination programme (at varying uptake rates) and are interested in adding boys to the schedule; whilst strategies 5 and 6 represent countries which are yet to begin vaccinating against HPV. In such a way, we showed how impacts changed depending on both the coverage of vaccination in the population and existing herd immunity.

### Economic model

The economic model took the form of a continuous time individual patient simulation (Additional file 2: Appendix S2), using output from the epidemiological model (specifically, times of infection and recovery, with each HPV type, for each individual in the model). The economic model then extrapolated these data to clinical events experienced by each individual over their lifetime (up to a maximum age of 100 years old). The healthcare costs and quality-adjusted life years (QALYs) for each vaccination strategy were compared to a baseline strategy (either no vaccination or girls only) and the incremental cost-effectiveness ratio of each strategy was estimated. We then generated the *threshold vaccine dose price*; that is, the maximum amount the healthcare system is willing to pay given the associated health benefits (currently set at £20,000 per QALY in the UK). Positive prices per vaccine dose below this threshold price will tend to generate positive net health benefits, whilst negative prices per vaccine dose offer no incentive to the manufacturer to provide the vaccine.

The economic evaluation was conducted from a UK National Health Service (NHS) and personal social services perspective with costs presented in pounds sterling (2013-14 prices). The following sections outline the basic clinical, cost and health utility parameters that fed into this economic model.

#### Clinical parameters

Age- and sex-specific incidences of the six cancer types included in the model (cervical, anal, vaginal, vulvar, penile, oropharyngeal), and cervical intraepithelial neoplasia were taken from 2013 UK cancer registration statistics. Age- and sex-specific incidences of genital warts were taken from a UK Health Protection Agency report [55], and age specific incidences of recurrent respiratory papillomatosis from a task force on recurrent respiratory papillomas [56].

Proportions of each of these clinical events associated with the HPV types included in the model were extracted from a published meta-analysis [57] and a literature review undertaken by Jit and colleagues [22] (Additional file 6: Table S4). It was often not possible to distinguish between events caused by types 6 and 11, so these were modelled as a single risk factor in the economic analysis. The same was true for events caused by types 31, 33, 45, 52 and 58.

Data on disease incidence, proportion of disease associated with HPV, and age- and sex-stratified proportions of people infected with each HPV type pre-vaccination were combined to give annual event rates for the nine diseases included in the model, stratified by age, sex and current and past HPV infection status.

All-cause mortality data were taken from the Office of National Statistics [58], as were age-specific one and five year survival data for cervical cancer [59, 60]. Data for other cancers were not available from the same source. Anal cancer survival rates were taken from an epidemiological study conducted by Jeffreys and colleagues [61], and those for other cancers from an Office for National Statistics report on survival rates from less common cancers [62]. However, since these data were old, the survival rates were adjusted to estimate contemporaneous values, using improvements in cervical cancer survival over the same time period. Mortality rates from recurrent respiratory papillomatosis were taken from a study by Bishai and colleagues [63]. For oropharyngeal cancer we use the proportion attributable to HPV from [64], and allow this proportion to increase up to 80% when sampling from economic parameters, to account for recent data [65].

#### Health utilities

Health utility decrements associated with cases of genital warts [66] and recurrent respiratory papillomatosis [63] were extracted from the literature. Health utility decrements associated with cancer consisted of two components, a short time loss during treatment, and a long term health utility decrement which persisted for the remainder of the individual’s life.

#### Costs

Costs of recurrent respiratory papillomatosis [67], genital warts [68], and cervical cancer [69] were all taken from the literature and inflated to 2013-14 UK prices. Costs for other cancer types were not available for the UK. Therefore, the relative costs (compared to cervical cancer) for these cancers were estimated from the HPV-ADVISE model [22], and these were indexed against the cost of cervical cancer in the model to obtain estimates of cost for other cancer types. The cost of vaccination administration was assumed in the baseline analysis to be £10 per dose (Department of Health, personal communication), and we assumed a two dose schedule (vaccine plus booster) following evidence that this is likely to be more cost-effective than three doses if vaccine protection is at least 20 years [29]. Given the high level of completion in the UK, we assumed for simplicity that all immunised individuals were given both doses, hence calculating the costs and impact of vaccination was straightforward. If a significant fraction of the population only received one dose, this might skew both the health impacts and the associated costs; however, this is not the case in the UK - in 2017/18, 83.9% received two doses while just 5.2% only received one dose and 10.7% did not receive any vaccine.

The costs and health utility decrements used in the model are summarised in Additional file 7: Table S5.

#### Time horizon and discounting

The time horizon of the base case model was 100 years post the point where the different vaccine strategies affected individuals in the model. Thus, people who were born at the start of 2000 were included in the analysis, as were all subsequent newborns.

To comply with the JCVI’s guidelines, two criteria were considered. Firstly, that for the most likely set of parameters (modes of posteriors) the mean discounted costs and outcomes should be evaluated against a £20,000 cost-effectiveness threshold value for a QALY [70]. Secondly, to account for uncertainty, 90% of all posterior parameters should generate cost-effective results at a threshold of £30,000 per QALY. In both cases discounting at a rate of 3.5% per year (for both healthcare costs and QALYs) was used.

As an alternative scenario, we also evaluated the effects of applying a 1.5% discount rate to health impacts; this was in response to the CEMIPP report [71] which highlighted that 3.5% discounting was not always appropriate given disparate delays from infection time to health effects, and alternative discounting rates should be considered where appropriate. This is the case for HPV, when there may be many years between vaccination, infection and the onset of life-threatening cancers. 1.5% was chosen in response to the appraisal by the National Institute of Care Excellence, which noted that “A discount rate of 1.5% for costs and benefits may be considered by the Appraisal Committee if it is highly likely that, on the basis of the evidence presented, the long-term health benefits are likely to be achieved” [70].

For the uncertainty criterion, we note that a single simulation contains stochasticity due to both parameter uncertainty, and also the finite size of the modelled population and the chance nature of transmission. Our results show that this second form of stochasticity is largely parameter invariant, and therefore we were able to separate these two effects (Supplementary Material). The results shown for the uncertainty analysis therefore reflect only our uncertainty in parameter estimates and not variability between simulations.

### Patient Involvement

Patient and public involvement (PPI) in research has become embedded in health research with patients and the public involved as collaborative partners through the research process [72, 73]. While common in health research, public involvement in mathematical and economic modelling is relatively rare, with few examples of embedded forms of collaborative involvement and a lack of agreed methodology for PPI in modelling.

For this analysis, patients and the public were not involved in developing either the research question or the design of the study in relation to the modelling approach, primarily because this is one of the first studies to include PPI in modelling. As such it is exploratory in nature, with our intention to identify the ways in which patients can contribute to modelling. We utilised the development of the HPV model as an opportunity to establish a PPI Reference Group (comprised of public members), to explore the potential for patients or the public to contribute to both the epidemiological and economic modelling components of the study, as part of the wider programme of work.

The Reference Group met regularly at key points in the study, with email contact in between. The wider aim of PPI within the project was to contribute towards conceptual development of PPI in mathematical and economic modelling, through the development of a new framework co-produced with patients and the public. Throughout the project we aimed to identify any impacts of the PPI Reference Group, and these will be disseminated through policy recommendations made by the Department of Health. A separate piece of work on the PPI contribution to the study is currently in progress.

## Results

The fitting scheme produced well-defined parameter distributions (see Additional file 8: Figure S1 and Table S6), and simulating using the distributions provided good agreement between the model and data (see Additional file 9: Figures S2 and S3). In the following sections the effects of varying vaccination strategy on HPV prevalence, incremental cost-effectiveness and the consequences for threshold vaccine dose prices, are presented.

### Epidemiological effects of vaccination strategies

**Fig. 1:**
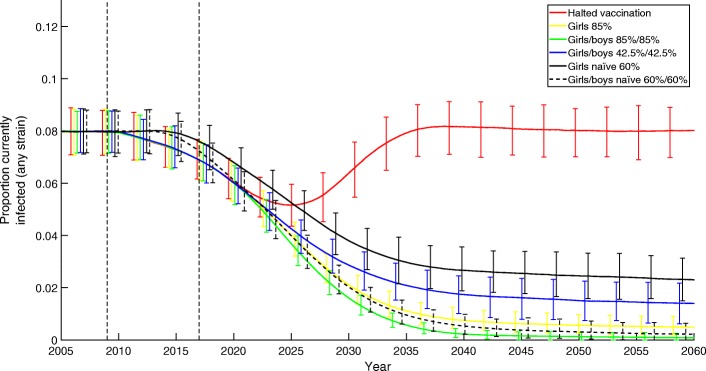
The effects of different vaccination strategies on the prevalence of HPV in the population, over the 30 years following a change in strategy. Strategies tested include: halted vaccination (red), girls only at 85% (yellow), girls/boys at 85%/85% (green), girls/boys at 42.5%/42.5% (blue), girls at 60% from 2008 (black solid), and girls/boys at 60%/60% from 2008 (black dashed). All strategies use the nonavalent vaccine

The predicted effect that a range of vaccination strategies would have on the prevalence of HPV is shown in Fig. 1.

The eight years of girls-only vaccination (2008-2016) had the effect of reducing HPV prevalence across the entire population (both male and female) from approximately 8% to 6.9%. Assuming that girls-only vaccination continues at 85%, by 2050 prevalence is predicted to drop to around 0.56% (yellow line). Adding boys’ uptake at 85% to the girls-only programme from 2017 onwards further reduces prevalence to approximately 0.13% (green line). As an alternative, keeping the number of vaccinations equal to the girls-only programme but splitting them equally between girls and boys (so that uptake is 42.5% in both sexes) leads to a less steep decline in prevalence, falling to around 1.5% by 2047 (blue line). Interestingly, halting vaccination entirely in 2017 leads to a continued fall in prevalence until 2025 (red line), due to the delay between vaccination and girls entering the sexually active population; however, in the longer term prevalence returns to approximately pre-vaccination levels.

As an alternative to the eight years of girls-only vaccination at high uptake, we investigated the effect of a lower uptake HPV vaccination campaign from 2008. Vaccinating just 60% of girls leads to a less marked decline in prevalence, reducing to around 2.5% by 2050 (black solid line); vaccinating 60% of both sexes further reduces the prevalence to 0.31% (black dashed line).

We note that, due to basing pre-2010 individual-level behaviour on Natsal-2 and post-2010 behaviour on Natsal-3 which reported marginally increased sexual behaviour [74], we observe slight increases in baseline prevalences from 2010 onwards. This can be seen most easily by contrasting prevalence pre-vaccination with values at 2050 for halted vaccination (7.97% and 8.02% respectively).

These results have two important public-health implications. Firstly the reduction in cases from adding boys to the vaccination program is markedly less that the initial impact of adding girls. Secondly, a gender-neutral campaign vaccinating 60% of the population has comparable impact on infection prevalence as vaccinating 85% of girls (42.5% of the population). Given the heterosexual nature of the majority of the UK population, it is clear that vaccination of girls is generating considerable herd-immunity for boys.

A detailed breakdown in the prevalence of different HPV strains, by age and gender, under alternative vaccination strategies is given in Additional file 11: Table S7. As might be expected, strains decrease according to the level of protection afforded by the vaccine as in Additional file 5: Table S3; for example, as strains 6 and 11 are not covered by the bivalent vaccine, prevalences of these strains are comparable between the strategies of halted, girls-only bivalent and gender-neutral bivalent vaccination. If the strain is covered by the vaccine, girls-only vaccination reduces strain prevalence significantly compared to halted vaccination, while adding boys to the girls-only programme has a more limited effect (e.g. for 26-35 year old males, strains 31/33/45/52/58 and the nonavalent vaccine, girls-only vaccination reduces prevalence from 10.2% to 2.05%; gender-neutral vaccination reduces prevalence to 0.658%, a lower incremental benefit).

### Cost-effectiveness of vaccination strategies

**Fig. 2:**
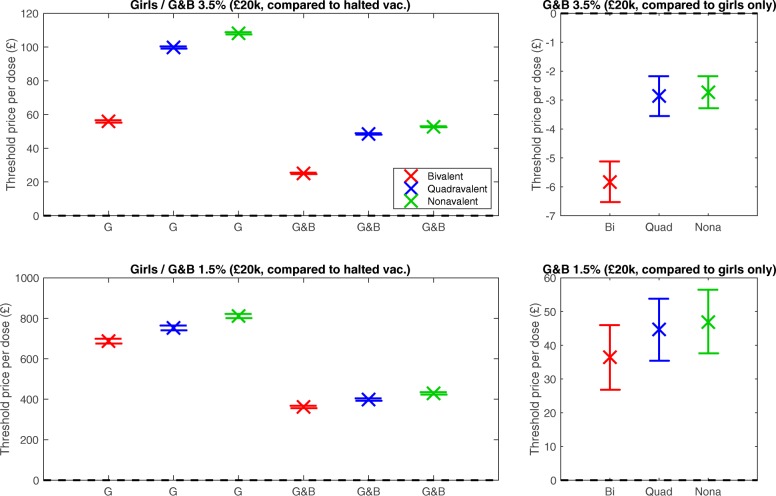
Threshold dose prices for various vaccination strategies, considering a two-dose schedule. All plots assume £20,000 cost-effectiveness threshold for a QALY, modal parameters from posterior used, and a £10 administration charge per dose. Mean values are shown as crosses, with 95% confidence intervals shown by bars. Colours correspond to the three vaccines: bivalent (red), quadrivalent (blue) and nonavalent (green). Top plots assume 3.5% discount rates applied, bottom plots assume 1.5% discount rates applied. Left plot: comparing girls-only and gender-neutral vaccination to halted vaccination. Right plot: comparing vaccination of gender-neutral vaccination to continuing girls-only vaccination. When comparing a strategy to girls-only vaccination the same vaccine is used for correct comparison

The mean results of the cost-effectiveness model are shown in Fig. 2 and Table 1, with the distribution of threshold prices shown in Additional file 10: Figure S4. Threshold dose prices for cost-effectiveness, using both 3.5% and 1.5% discounting, with a £20,000 cost-effectiveness threshold value, are shown for all vaccination strategies versus halted vaccination, and for gender-neutral strategies versus girls only. Prices are shown for the cost per dose, assuming a two-dose schedule, and a £10 administration charge for each dose.

**Table 1:** Threshold dose prices for various vaccination strategies, considering a two-dose schedule

Vaccination strategy	£, versus halted vac. (3.5%)	£, versus girls’ vac. (3.5%)	£, versus halted vac. (1.5%)	£, versus girls’ vac. (1.5%)
Girls, bivalent	55.80 (55.04 – 56.57)	-	687.47 (675.62 – 699.20)	-
Girls, quadrivalent	99.64 (98.90 – 100.37)	-	752.46 (740.51 – 764.72)	-
Girls, nonavalent	108.05 (107.36 – 108.75)	-	811.44 (801.18 – 821.68)	-
Girls & boys, bivalent	25.08 (24.64 – 25.52)	-5.67 (-6.42 – -4.93)	361.99 (356.09 – 367.85)	36.46 (26.82 – 45.98)
Girls & boys, quadrivalent	48.38 (47.95 – 48.80)	-2.92 (-3.64 – -2.18)	398.68 (392.65 – 404.84)	44.70 (35.42 – 53.81)
Girls & boys, nonavalent	52.77 (52.42 – 53.10)	-2.56 (-3.13 – -1.99)	429.26 (422.98 – 435.44)	46.88 (37.63 – 56.46)

Mean values shown over 500 simulations, with 95% confidence intervals in brackets. All strategies assume a £20,000 cost-effectiveness threshold for a QALY, with both 3.5% and 1.5% discount rates applied, and a £10 administration charge per dose. When comparing a strategy to girls-only vaccination the same vaccine is used for correct comparison

Vaccinating girls only or both girls and boys, with any of the vaccines, was always cost-effective compared to not vaccinating, with positive threshold dose prices and positive confidence intervals in all instances. However, vaccinating girls alone was more cost-effective per dose, with a higher threshold price for each vaccine, compared to a gender-neutral strategy. Generally, the nonavalent vaccine was the most effective in preventing disease, followed by the quadrivalent, and finally the bivalent, as would be expected from the level of protection offered, hence a greater threshold price. Incremental to a girls-only vaccination campaign, adding boys gave threshold dose prices very close to, but below, zero, at 3.5%. The results from individual simulations varied widely and 500 replicates were needed to achieve relatively tight confidence intervals around the mean. For the quadrivalent vaccine, the mean dose price was negative (at -£2.92) and, given that the confidence intervals are below zero (from -£3.64 to -£2.18), we can say with 95% confidence that the threshold price is negative. The same arguments apply to both the bivalent and nonavalent vaccines.

At 1.5% discounting all threshold prices increased, but with the same qualitative patterns; for girls-only vaccination compared to halted vaccination threshold prices were £687 – £811 for the three vaccines and gender-neutral vaccination was less cost-effective, with threshold prices of £362 – £429. Incremental to girls only, gender-neutral vaccination had positive threshold prices of £36 – £47. This is due to the lower discount rate adding more weight to economic values placed on health conditions in the future. In general, a lower discounting rate will always make vaccination more cost-effective for infections like HPV, where the health consequences are experienced years or decades after infection.

A detailed breakdown in the reduction in cases of the health sequelae under alternative vaccination strategies is given in Additional file 12: Table S8. As might be expected, there is a large decrease in cases of health sequelae when vaccinating girls compared to halted vaccination, while adding boys to the girls-only programme yields a much smaller decrease in cases. Incremental cost-effectiveness ratios (ICERs) for alternative vaccination strategies are shown in Additional file 13: Tables S9 and S10, and Figure S5, at a range of assumed vaccine dose prices. Relative to halted vaccination, girls-only vaccination results in lower ICERs than a gender-neutral programme (Additional file 13: Tables S9), while ICERs are significantly higher for a gender-neutral programme compared with girls-only vaccination, signalling low cost-effectiveness (Additional file 13: Tables S10).

**Fig. 3:**
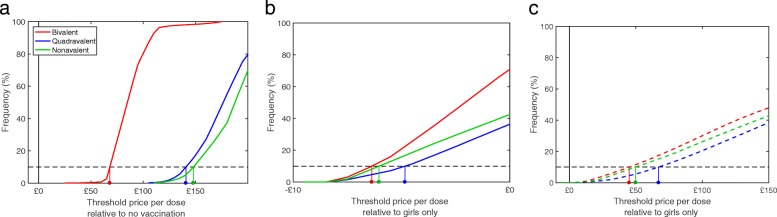
Threshold dose prices for girls-only vaccination compared to halted vaccination (at 3.5% discounting, left plot), and gender-neutral vaccination compared to girls-only vaccination (at 3.5% discounting, middle plot, and at 1.5% discounting, right plot). Coloured lines show different vaccines: bivalent (red), quadrivalent (blue) and nonavalent (green). Threshold dose prices at the 10th percentile of simulated values highlighted by coloured lines from x-axis to curve. Vertical black dashed line indicates £0 threshold price. Results shown for 500 simulations

Employing the probabilistic approach as per the JCVI’s guidelines, whereby parameters in both the transmission and economic models were sampled from appropriate distributions, and increasing the cost-effectiveness threshold for a QALY to £30,000, the threshold dose prices at the 10th percentile of simulated values (for bivalent, quadrivalent and nonavalent vaccines, and at both 3.5% and 1.5% discount rates) are shown in Table 2, and the cumulative distribution of threshold dose prices are displayed in Fig. 3.

**Table 2:** The threshold dose prices at the 10th percentile of simulated epidemiological and economic values (over 500 simulations), using both 3.5% and 1.5% discount rates and an increased cost-effectiveness threshold of £30,000, compared to both halted vaccination and girls-only vaccination, with a £10 administration charge per dose

Vaccination strategy	£, versus halted vac. (3.5%)	£, versus girls’ vac. (3.5%)	£, versus halted vac. (1.5%)	£, versus girls’ vac. (1.5%)
Girls, bivalent	68.07	-	1024.71	-
Girls, quadrivalent	140.85	-	1121.74	-
Girls, nonavalent	147.90	-	1232.70	-
Girls and boys, bivalent	32.13	–6.38	577.45	45.06
Girls and boys, quadrivalent	74.95	-4.84	654.07	67.12
Girls and boys, nonavalent	81.12	-6.04	676.54	49.89

It is evident that, while vaccination of girls fulfilled the JCVI criterion of 90% of simulations generating cost-effective results (Fig. 3a), adding boys to an existing girls-only campaign at 3.5% discounting did not satisfy the condition (Fig. 3b). For all three vaccines, the 10% boundary in the cumulative distribution for threshold dose price lay below zero - meaning that, by the JCVI guidelines for uncertainty, adding boys is not cost-effective - further supporting the conclusion from the results using the most likely parameters.

Conversely, at 1.5% discounting (Fig. 3c) all three vaccines had positive values at the 10% boundary (£45 – £67). Hence at 1.5% both criteria for cost-effectiveness are met. We note that the 10% boundary price for the quadrivalent vaccine is higher than for the nonavalent (at both 3.5% and 1.5% discounting). We suggest this is because vaccinating girls only with the nonavalent vaccine induces herd immunity against all 9 HPV types such that the addition of vaccinating boys has limited impact; in contrast, given that the quadrivalent vaccine induces only limited herd immunity against types 31, 33, 45, 52 and 58, including vaccination of boys has a more substantial impact on these types.

**Fig. 4:**
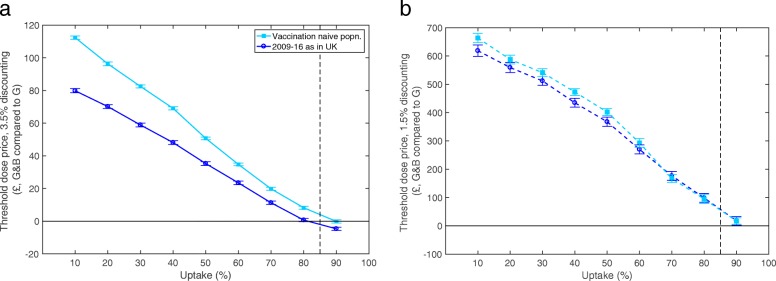
The mean threshold vaccine dose price for gender-neutral vaccination compared to girls-only vaccination, using the quadrivalent vaccine, for **a** 3.5% discounting and **b** 1.5% discounting. Two initial conditions are tested: assuming no vaccination has previously occurred (square markers), or assuming uptake in girls as in the UK historically for 2008-2016 (circle markers). 95% confidence intervals shown for all points. Vertical dashed line highlights 85% uptake, which is the rate assumed for girls-only and gender-neutral vaccination in the future in this paper. 200 simulations carried out for each data point shown

Considering the different levels of vaccine uptake in more detail and the resulting herd-immunity provides a richer understanding of the cost-benefit relationship (Fig. 4). Two elements contributed to the low threshold dose price previously described. The first is that, as the uptake rate of vaccination in both boys and girls increased, so the threshold dose price decreased; this is because of the herd immunity generated by immunising an increased proportion of girls such that much of the vaccination in boys was effectively “wasted”, i.e. the majority of men who were vaccinated as adolescents will not be subsequently exposed to infection. The second is that the high vaccination uptake rates observed so far in the UK, have already generated such high levels of population protection that the impact of vaccinating boys was further reduced (Fig. 4, circle markers compared to square markers). Thus, if uptake is expected to be low across a population, then introducing a gender-neutral scheme early is expected to be highly beneficial; for example, at 3.5% discounting and assuming uptake was just 10%, the mean threshold price for adding boys was £112.37 (111.35 - 113.40) without prior vaccination or £78.11 (76.60 - 79.58) if girls had been vaccinated for eight years as assumed elsewhere in this paper.

We note that, using 3.5% discounting (Fig. 4a), at 85% uptake (dashed vertical line) the cost-effectiveness of gender-neutral vaccination drops below zero for a population with UK vaccination up until present, whereas it is positive for a vaccination-naïve population. As with the previous simulations, decreasing the discounting rate to 1.5% increases all threshold prices (Fig. 4b), with positive prices up to and including 90% uptake rates.

## Discussion

The modelling work performed here combined epidemiological and economic insights with advice from our PPI group, and provides cost-effectiveness results for a variety of vaccination strategies to combat HPV, following standard methodologies (e.g. [19, 26, 75–77]. Previous studies have predicted that vaccination of girls would be highly cost-effective [19, 26, 75], with concomitant decreases in levels of HPV infection (and associated adverse health sequelae) [9, 10, 78–80]. The majority of previous economic analyses indicated that adding boys to the girls-only programme is unlikely to be cost-effective (e.g. [24, 26, 43, 45, 81]. The results presented here echo these findings, but provide both a UK-specific context and a broader scientific understanding of the impact of vaccine uptake and economic discounting.

Our fitted epidemiological model provides a good match to the available datasets. The use of likelihood-based techniques and Bayesian MCMC methodology meant that we could account both for inconsistencies between data sets and uncertainty in parameter estimates. In particular, while parameter estimates for the main HPV types (6, 11, 16 and 18) are well defined, there is greater uncertainty surrounding the additional five types in the nonavalent vaccine (31, 33, 45, 52 and 58), reflecting the sparsity of data sources (Additional file 9: Figures S2 and S3). A lower probability of serological response in men compared to women is in agreement with a recent meta-analysis on natural immunity [82]; although that study found immune period difficult to determine due to a lack of knowledge of infection time, our model suggests a period of around 1.4 years.

Vaccination of girls has been observed to lower the population prevalence of HPV [11, 80], and our models suggest that in the UK this trend is likely to continue (Fig. 1, yellow line). Even if vaccination is stopped, the time-delay between vaccinating young girls and them entering the sexually active population means that prevalence will continue to fall for another nine years before finally returning to pre-vaccination levels of around 9.5% (for any of the nine types modelled here). A girls-only vaccination campaign with 85% uptake with the nonavalent vaccine is expected to reduce population prevalence to around 1% within forty years. This value is further reduced to just 0.2% if boys are also included in the vaccination scheme. Therefore, we find that while introducing vaccination in young girls leads to a reduction in prevalence of around 90%, adding boys to this scheme only provides a limited further reduction in prevalence. Given that the majority of HPV transmission is through heterosexual relationships (so the infection must pass through a male-female-male-female …chain), it is clear that completely protecting either sex is sufficient to halt heterosexual transmission. This helps to explain why adding boys to an effective girls-only programme has limited effect. The exception to this is MSM, where there is limited herd-immunity from the girls-only programme, and so vaccination could be expected to be highly effective [34, 35, 47]. However, a mass (i.e. untargeted) vaccination programme of boys does not target this group.

In general, any additional vaccination will always reduce the prevalence of infection and hence the expected amount of disease; furthermore, greater reductions are naturally predicted for vaccines that provide protection against more HPV types. To understand whether these declines are worth the additional costs of vaccination, requires us to undertake a health economic evaluation comparing health benefits against vaccine-related costs and associated costs. All tested strategies were cost-effective (at the £20,000 cost-effectiveness threshold for a QALY and assuming 3.5% annual discounting) compared to halted vaccination. Girls-only vaccination was highly cost-effective versus halted vaccination, with threshold dose prices of £55.80, £99.64 and £108.05 for the bivalent, quadrivalent and nonavalent vaccines, respectively (Table 1). Gender-neutral vaccination was also cost-effective versus halted vaccination, although at lower threshold dose prices of £25.08, £48.38 and £52.77 for the three respective vaccines. All threshold prices were higher at 1.5% discounting, but the same qualitative pattern observed (Table 1).

Comparing gender-neutral vaccination with girls-only vaccination (that is the cost-effectiveness of adding boys), none of the vaccines had a positive dose threshold price at 3.5% discounting (-£5.67, -£2.92 and -£2.56 for the three vaccines), with confidence intervals that were all below zero (Table 1). Moreover, following UK guidelines for economic evaluations of immunisation programmes, we examined the uncertainty in our predictions. Even removing between-simulation stochasticity, the uncertainty in parameter estimates was such that there is a less than 10% chance that gender-neutral vaccination is cost-effective (compared to girls-only) at a cost-effectiveness threshold value for a QALY as high as £30,000 (Fig. 3b). In contrast, when comparing all strategies to halted vaccination, the recommended probabilistic threshold is always achieved (Table 2).

At 1.5% discounting all threshold dose prices increase, and gender-neutral vaccination, incremental on girls-only vaccination is cost-effective, with threshold prices of £36.46, £44.70 and £46.88 (Table 1). There is also a greater than 90% chance that gender-neutral vaccination is cost-effective (compared to girls-only) at a cost-effectiveness threshold value of £30,000 per QALY (Fig. 3c). Recent recommendations from the CEMIPP report [71] and the JCVI [83] suggest applying a lower discount rate to the health effects of HPV vaccination, given the long time delay between infection and the onset of adverse sequelae.

There is some empirical evidence of cross-protective effects of the bivalent vaccine against warts-causing types 6 and 11 [10, 84]. Although we have not tested the assumption here, the fact that the quadrivalent and nonavalent vaccines (which offer complete protection against 6 and 11) are not cost-effective for a gender-neutral programme at 3.5% discounting, means that adding these cross-protective effects will not change the conclusions reached.

As vaccine coverage in girls increases, so herd-immunity builds up, making additional vaccination less worthwhile. Hence, with higher uptake rates in girls (and longer historical vaccination of girls), boys’ vaccination becomes less cost-effective (Fig. 4). This is a critical result, as it shows that in the UK, where uptake in girls has historically been so high [11, 54] adding boys is not a cost-effective option. Conversely, in other countries where existing girls-only programmes have lower uptake rates (such as the USA and France [13]) adding boys may indeed still be cost-effective. This is due to the higher prevalence of infection remaining in the population, leading to potentially more significant declines in the incidence of adverse health effects such as cervical and oropharyngeal cancer. Similarly, including boys in the vaccination scheme can buffer the programme against fluctuations in level of vaccine uptake [85]. This impact of uptake in girls, echoes previous findings. Given the significant variation in uptake of the girls’ programme between countries [12], we would expect to observe a threshold below which gender-neutral vaccination was economically acceptable; however, the reality is more ambiguous. In particular, Australia, New Zealand and Canada have recently added boys to their respective national vaccination programmes, and uptake rates are 71% [13], 40-56% [13, 15] and 39-89% [86, 87] in these respective countries. For New Zealand this contradicts a recent study which found that adding boys would not be cost effective [43]. Clearly, these health economic arguments are strongly influenced by the vaccine price, with recent reductions in average tender prices favouring the adoption of a gender-neutral programme [88].

Girls carry a larger economic burden of HPV-related disease than boys [89], due to the relatively high incidence of cervical cancer, so they are always the primary target for vaccination. This is evidenced in Additional file 12: Table S8, where the incidence of cervical cancer with halted vaccination is higher than all cancers except for oropharyngeal cancer. The addition of boys’ vaccination gives small reductions in prevalence of these health effects, in comparison to vaccinating girls alone. Combining the incidence rates in Additional file 12: Table S8 with the costs and QALY decrements in Additional file 7: Table S5, we calculate the main cost-saving impact of adding boys is reductions in oropharyngeal cancer and genital warts, with cervical cancer reduction having reduced savings. In low uptake countries, questions remain as to whether it would be more cost-effective to vaccinate boys or to try to increase the coverage in girls, with more studies suggesting the latter [39]. Recent work, however, has highlighted the increasing importance of other cancers as HPV vaccination programmes lead to declines in cervical cancer cases [90].

It is clear from our analysis that mixing patterns are an important factor in the spread of HPV. An aspect not explicitly modelled here was the possibility of disease import from outside the population (i.e. immigration and tourism). This has been considered in some of our work, but unprotected sex with unvaccinated individuals from outside the UK is likely to be a relatively minor component [91]. It is also likely to be highly non-random in a way in which there is little data to support any assumption.

One limitation of our modelling approach is the decoupling of HPV vaccination from cervical cancer screening – we have implicitly assumed throughout this work that cervical cancer screening will continue in its current form. It is possible that the nature of (and hence costs and consequences of) the cervical cancer screening programme will change in the future. In the near term this is most likely to be caused by evidence showing HPV-based cervical screening is more effective than the current cytology-based programme, and supports increasing the screening intervals from three to five years [92]. A programmatic change to primary HPV testing for cervical cancer is predicted to reduce cervical cancer incidence [93]; this has the potential to reduce the estimated cost-effectiveness of HPV vaccination in the UK. On the other hand, screening programmes could change directly as a result of the HPV vaccination programme; it has been estimated that the successful use of the bivalent vaccine could reduce the need for more than three cervical smears per lifetime [94]. A recent study from Australia concluded that, if continued gender-neutral vaccination was maintained into the future, cases of cervical cancer could reduce from seven cases per 100,000 women to less than four by 2028 [95]. Overall, while the current cervical screening programme is implicitly accounted for in our study (ignoring any possible fluctuations in screening participation by year/age/vaccination status), we do not account for the impact of future changes to screening technology, interval and compliance. Thus, far more research is needed to inform best policy on the interaction between HPV vaccination and cervical cancer screening. Future changes to the cervical cancer screening programme are beyond the remit of this study, but are an important area of focus for future research.

A key assumption in our models, which may require further study is that vaccination is an independent random process, and in particular is not correlated with sexual behaviour. If this is not appropriate, it may be that the girls who are missing out on vaccination are in the highest risk groups, and may be disproportionately contributing to transmission of HPV. Due to the high prevalence of HPV across both men and women [96–98], this seems unlikely to have a significant effect on the dynamics of infection, although other modelling studies have shown that the correlation does not need to be big to have an impact [99]. Whilst there is no clear consensus of drivers of vaccine uptake at a community-level [12], there is more evidence at the individual level of a relationship between vaccination and risk behaviour [100, 101]. When risk is highly clustered to particular groups, for example for hepatitis-B, then the ability to target these groups becomes critical to the decision to vaccinate [102]. An important follow-up to this work would be to assess the importance of low- and high-risk infection groups, their likelihood of vaccination, the mixing between the two groups, and the effectiveness of vaccination given the relative coverage in the high-risk group.

## Conclusions

The generic conclusion from this work is that as coverage in girls increases, there is less incremental benefit from adding boys to the programme, due to existing herd-immunity. In the case of the UK, with the highest reported sustained HPV vaccine uptake rates in girls of any country, it is unlikely that adding boys will be cost-effective within standard economic guidelines which assume a 3.5% economic discounting. However, given the long time-scales associated with HPV infection and resulting disease, it may be more appropriate to adopt a 1.5% discounting, in which case adding boys to the programme becomes cost-effective for all three vaccines considered.

## Additional files

Additional file 1 Appendix S1. A detailed overview of the key assumptions underpinning the epidemiological model employed in the paper. (PDF 70 kb)

Additional file 2 Appendix S2. Economic model assumptions. (PDF 40 kb)

Additional file 3 Table S1. A summary of the datasets used to fit the HPV transmission model to for pre-vaccination populations. (PDF 53 kb)

Additional file 4 Table S2. Vaccine uptake in the UK, 2008–2016. (PDF 746 kb)

Additional file 5 Table S3. Efficacy of the three HPV vaccines against different HPV types. (PDF 48 kb)

Additional file 6 Table S4. Clinical parameter values and sources. (PDF 50 kb)

Additional file 7 Table S5. Costs and health utility decrements. (PDF 55 kb)

Additional file 8 Figure S1 and Table S6. Parameter distributions. (PDF 176 kb)

Additional file 9 Figures S2 and S3. Comparing HPV prevalence between the model and data. (PDF 328 kb)

Additional file 10 Figure S4. The mean threshold price per dose, under different vaccination strategies, for the base case scenario, at both 3.5% and 1.5% discount rate. (PDF 154 kb)

Additional file 11 Table S7. The mean prevalence of different HPV strains (as a percentage) in different ages and genders, after 50 years of simulating a range of vaccination strategies. (PDF 51 kb)

Additional file 12 Table S8. Cases of disease for different vaccination strategies. (PDF 1254 kb)

Additional file 13 Tables S9 and S10, and Figure S5. Incremental cost-effectiveness ratios. (PDF 146 kb)

## Abbreviations

HPV: Human papillomavirus
ICER: Incremental cost-effectiveness ratio
JCVI: Joint Committee on Vaccination and Immunisation
MCMC: Markov Chain Monte Carlo
MSM: Men who have sex with men
NATSAL: National Survey of Sexual Attitudes and Lifestyles
NHS: National Health Service
PPI: Patient and public involvement
QALY: Quality-adjusted life year
SIRS-V: Susceptible - Infected - Recovered - Susceptible - Vaccinated
UK: United Kingdom
