# Supplementary material for: Assessing the cost-effectiveness of HPV vaccination strategies for adolescent girls and boys in the UK
Source: BMC Infect Dis. 2019 Jun 24;19:552. doi: 10.1186/s12879-019-4108-y (PMC6591963; doi:10.1186/s12879-019-4108-y)
Supplement: Supplementary file 1 — Appendix S1. A detailed overview of the key assumptions underpinning the epidemiological model employed in the paper. (PDF 70 kb) [file 12879_2019_4108_MOESM1_ESM.pdf]

Additional Files

Additional file 1 — Appendix S1

A detailed overview of the key assumptions underpinning the epidemiological model employed in the paper.

- We assume SIRS-V (Susceptible - Infected - Recovered - Susceptible - Vaccinated) dynamics - individuals start life susceptible to all HPV types, may become infected with one or more types as sexual partnerships are formed, can transmit infection to others in subsequent partnerships, and then naturally recover from infections after some time. Temporary natural immunity (entering the recovered class) is assumed after clearing an infection, although eventually individuals move from recovered back into the susceptible class. Vaccination moves individuals from any of the S, I or R classes into a vaccinated class, where infection is highly unlikely; after a period of time protection wanes.
- We assume that natural infection confers temporary immunity on an individual, and there is waning of this immunity after some time (a parameter δ is fitted to the data). Following this, full susceptibility returns, and initial infections are no more or less likely than subsequent infections.
- We assume that vaccination effectively treats and immunises infected individuals - an assumption which would impact catch-up vaccination programmes in older cohorts where there may be high prevalence of HPV, but is unlikely to change the results for routine vaccination of 12-13 year olds.
- Nine types of HPV (types 6, 11, 16, 18, 31, 33, 45, 52 and 58) are modelled and the types are assumed to be independent (i.e. having one type does not make infection by another type more or less likely). The impact of this independence assumption is unclear: if there is within-host competition between the types, then immunisation against the common types (6, 11, 16 and 18) may lead to other types dominating, weakening the impact of vaccination; in contrast, if infection with one type leaves individuals more susceptible to other types, this impact will be reversed. In the absence of any data on type interaction, we made the simplest assumption that the types transmit independently.
- We fit the model to recorded prevalence of the nine HPV types using differences in transmission probabilities; an alternative mechanism would be to use differences in infectious period or a mixture of the two. Initially, we attempted to fit both transmission probability and infectious period for each type, but found the inferred values of these two parameters to be highly variable yet highly correlated. Dynamically the impact of these two parameters is indistinguishable (prevalence is largely determined by the basic reproductive ratio which is a product of transmission rate and infectious period). In discussions with HPV clinical experts, it was felt that having type-specific transmission probabilities was more informative, as it was unclear how detectable HPV infection relates to the ability to transmit. Previous work has shown rate of recovery from infection to be an important determinant of type-specific herd effect ([103, 104]), although these studies assumed the same transmission rate across all types. However, the meta-analysis of Rositch et al. ([104]) shows there is huge variability in the duration of infection as measured by the different studies. Furthermore, patterns are not always consistent; Rositch et al. ([104]) have the longest median duration of infection for strain type 31, whereas Lehtinen and Dillner ([103]) have measured this as comparable to other medium duration types (18, 33 and 52). In general, we believe that both the predicted public-health impact and the predicted economic impact of HPV is unaffected by whether we fit to the infectious period or the transmission probability.
- The data used to underpin the epidemiological component of this study, came from a detailed literature search for studies reporting both age- and type-structured prevalence data from the pre-vaccination era. The choice of these datasets was refined and ratified by an independent expert group. By using Bayesian parameter inference, we were able to find model parameters which maximised the likelihood of matching to all data sets, accounting for sample sizes. We note that, while many of these datasets come from outside of the UK, we felt that the benefits of having the additional sample size and information compensated for the lack of UK-specificity. In a similar manner we use both serological and DNA prevalence data, by accounting for seroconversion.
- We simulate using an individual based stochastic model, with the number of individuals generally picked to be 50,000 for simulations. This population size was a compromise between computational efficiency and lack of stochastic variation; larger population sizes produced less variable results but took far longer to simulate. Prior testing of different population sizes confirmed that population sizes of these magnitudes are

not influencing our results. Immigration of individuals and tourism (and hence introduction of HPV) are not modelled, as it is difficult to quantify the amount of HPV being introduced to the UK in this manner ([91]).

- Individuals are defined by four factors: age (10-50), sex (male and female), sexual preference (heterosexual or homosexual/bisexual) and the percentile of the distribution of expected partnership rates. This percentile, together with the other three defining factors, is used to determine the rate at which new partnerships are formed.
- Partnership rates and age of partners are taken from the Natsal-2 and -3 datasets (the former until 2010, the latter from 2010 onwards) - this comprises of behaviour and choice of partner dependent on age, sex and sexual preference of individuals ([105, 74]). Although HPV urine tests were carried out on a large sample of individuals in Natsal-3 ((all 16-17 year olds; all 18-24 year olds who reported at least one sexual partner, ever; a random sub-sample of 85% of 25-44 year olds who reported at least one sexual partner, ever; and any remaining 25-44 year old men who reported having sex with another man in the past five years)) ([79]), we chose not to include them as information for our model fit. This was because the reliability of the urine test for assessing HPV prevalence is low compared to either seropositivity and DNA tests from other anatomical sites.
- Individuals who have not yet had a sexual partnership have separate rates of forming their first sexual partnership, which are determined from Natsal.
- As individuals age, their partnership rates change to reflect national patterns; in addition, individuals randomly change their partnership rate percentile to account for dramatic changes in behaviour. In other words, the distribution of partnership rates changes with age from the Natsal data; in addition, as people become a year older, we resample the percentile values for a subsample of the population, so that varying behaviour with age is possible. In such a way we account for switching between having multiple sequencial partnerships and forming a long-term partnership (and vica versa) which occurs within the population.
- We do not directly model the making and breaking of partnerships; instead we assume that the formation of a new partnership is always preceded by the breaking of the old partnership. We assume that new partners are chosen from an independent pool of the population at the partnership rate of the individual, with the infection status of this external pool mirroring the dynamics within the focus population. Thus, we do not explicitly pair up individuals in the population, but rather assume that the different individuals are representative of the wider population, and when a partnership is made, if the chosen partner has any of the HPV types, there is a probability of the partner from the focal population catching the type(s). This is based on evidence that transmission of HPV infection within relationships appears to be fast. This formulation for the dynamics of partner formation and transmission is explored in more detail in Datta et al. ([50]).
- We note that concurrency is not explicitly modelled here, i.e. if person A is in a partnership with person B, then begins a new partnership with person C and is infected with one or more types, these cannot be transmitted to person B. Information from Natsal-3 ([106]) indicates that concurrency is not uncommon and could potentially alter the dynamics. However, results from simpler model structures suggest that once models parameters are inferred to match prevalence data, there is limited impact from concurrency within the population ([107]).
- For sexual partnering behaviour, a parameter (assortativity) is introduced for the likelihood of partnering an individual with a similar partnership rate to one's own (i.e. high partnership rates with other high partnership rate individuals); we fit this by calculating the effect of the assortativity on the HPV prevalence across age groups (i.e. how mixing of different partnership rates increases the spread of HPV).
- We include a parameter for the ratio of outward male transmission to outward female transmission, due to data suggestive that there are noticeable differences between the two sexes ([51]), and use prevalence data available on both sexes to fit this ratio. We find that there is a slightly higher rate of transmission from males compared to transmission from females, although the result is not statistically significant.
- In terms of HPV type dynamics, when a new partnership is established, infecting HPV types are transmitted from the partner in the wider population to the partner from the focal population with independent probabilities (we may therefore get no transmission, transmission of just one infecting type or multiple transference). Intensity of sexual contact or types of sexual act are not modelled, as there is relatively little data available to parameterise the risks associated with this additional heterogeneity.

- With each infection by a strain we assume a probability of seroconversion (i.e. production of antibodies against the HPV strain), which is disaggregated into male and female seroconversion, due to evidence of differing rates ([96], [82]). We fit these two parameters to the serological datasets included in Additional file 3 — Table S1. Note that we assume instant seroconversion in the model, whereas some studies show a delay between DNA presence and antibody production (e.g. 12 months [96]).
- We model the waning of vaccine-induced immunity as a sigmoidal function, centred at 20 years such that protection against vaccine types is high leading up to 20 years, but then wanes, and is close to zero by 25 years. This assumption comes from the latest HPV vaccination data and the expert opinion of immunologists on the JCVI panel. This applies to all three vaccines modelled (bivalent, quadrivalent and nonavalent). We assume that the nonavalent vaccine protects against all nine types equally, and efficacy is regardless of prior or current infections. As for cross-protection, we use values from ([108]) to inform our model, with numbers as in Table S3. (Values from ([22]) were also considered, but the type-specific detail in the former article made it the more appropriate). All cross-protection wanes at the same rate as for the vaccine types (almost total for 20 years, before dropping off sigmoidally to zero by 25 years). Alternative patterns of waning immunity are possible and have been assumed in other work; for example, the simplest assumption of exponentially decaying immunity will decrease the impact of immunisation, due to the proportion of individuals who lose protection between vaccination and becoming sexually active.
- We assumed the same levels of induced protection by the vaccine in both boys and girls, and the same rate of waning immunity. We also assume that a 2-dose schedule (at 0 and 12 months) is equally protective as a 3-dose schedule (at 0, 6 and 12 months); see Additional file 5 — Table S3.
