# Supplementary material for: Assessing the cost-effectiveness of HPV vaccination strategies for adolescent girls and boys in the UK
Source: BMC Infect Dis. 2019 Jun 24;19:552. doi: 10.1186/s12879-019-4108-y (PMC6591963; doi:10.1186/s12879-019-4108-y)
Supplement: Supplementary file 2 — Appendix S2. Economic model assumptions. (PDF 40 kb) [file 12879_2019_4108_MOESM2_ESM.pdf]

Additional file 2 — Appendix S2

Economic model assumptions.

In the following section we provide a detailed overview of the economic model employed in the paper.

- Economic costs are based on the results of individual-level discrete event simulations, in which the lifetime health consequencies (related to HPV infection) are modelled for 100,000 individuals over the simulated time horizon.
- Individuals have a maximum age of 100 years old, but have death rates which are age-dependent and are based on the latest UK estimates.
- HPV associated events included in the economic model are genital warts, recurrent respiratory papillomatosis, and six types of cancer (CIN/cervical, vaginal, vulvar, anal, penile and oropharyngeal). Each of these has associated economic and health costs.
- An individual's risk of developing one of these events, at any point in time, is determined by their age, current HPV infection status and which HPV types they have been infected with in the past. However, the risk of disease is assumed independent of number of previous HPV infections with the same type, time since infection (provided they are not currently infected), and age of initial infection. Changes to these basic assumptions are likely to increase the cost-effectiveness of vaccination, as both infection times and hence disease event times are pushed later into an individual's lifetime.
- HPV infections are assumed to change only the probabilities of initially developing each of the different health sequelae, and not the speed with which that sequela progresses once it has developed, nor the final outcome of the sequela.
- The effects on event probabilities by types 6 and 11 are modelled jointly rather than separately, and the same for HPV-16 and 18. This is because most studies that report the proportion of various cancer types associated with HPV infection report jointly for the two types, rather than separately.
- There are no direct costs or health-related quality of life losses associated with HPV infection itself; only through the events it later causes.
- The costs associated with sequelae of HPV infection are all taken from UK studies, as we expect these to be country-specific.
