# Supplementary material for: Assessing the cost-effectiveness of HPV vaccination strategies for adolescent girls and boys in the UK
Source: BMC Infect Dis. 2019 Jun 24;19:552. doi: 10.1186/s12879-019-4108-y (PMC6591963; doi:10.1186/s12879-019-4108-y)
Supplement: Supplementary file 3 — Table S1. A summary of the datasets used to fit the HPV transmission model to for pre-vaccination populations. (PDF 53 kb) [file 12879_2019_4108_MOESM3_ESM.pdf]

Additional file 3 — Table S1

A summary of the datasets used to fit the HPV transmission model to for pre-vaccination populations.

No.	Data	Country	Data type	Sex (number)	Age groups	Types included
1	Desai et al., 2011 ([96])	England	Sero+	M (2400), F (2400)	10-49 separate	6, 11, 16, 18
2	Introcaso et al., 2014 ([109])	USA (NHANES 2003-2006)	Sero+	M (3673), F (3956)	14-19, 20-24, 25-29, 30-39, 40-49	6, 11, 16, 18
3	Hariri et al., 2008 ([110])	USA (NHANES III)	Sero+	M (2644), F (3455)	12-19, 20-29, 30-39, 40-49	11
4	Stone et al., 2002 ([111])	USA (NHANES III)	Sero+	M (2752), F (3580)	12-19, 20-29, 30-39, 40-49	16
5	Jit et al., 2007 ([112])	England	Sero+	F (1483)	10-29 separate	6, 11, 16, 18
6	Ryding et al., 2008 ([81])	Sweden	Sero+	M (1383), F (1709)	11-19 separate, 20-22, 23-26	16
7	Castro et al., 2014 ([113])	Chile	Sero+	F (268)	15-20, 21-30, 31-40, 41-50	16, 18, 45
8	Sargent et al., 2008 ([114])	England (ARTISTIC)	DNA	F (18860)	20-29, 30-39, 40-49	16, 18, 31, 33, 45, 52, 58
9	Tanton et al., 2017 ([49])	England (Natsal-3)	DNA	sex. active F (3282)	16-29	6, 11, 16, 18, 31, 33, 45, 52, 58
10	Giuliano et al., 2008 ([115])	USA	DNA	M (290)	18-44	6, 11, 16, 18, 31, 33, 45, 52, 58
11	Kavanagh et al., 2008 ([116])	Scotland	DNA	F (2062)	20-21	16, 18, 31, 33, 45, 52, 58
12	Nielson et al., 2007 ([48])	USA	DNA	sex. active M (463)	18-40	6, 11, 16, 18, 31, 45, 52
13	King et al., 2015 ([35])	England	DNA	MSM (511)	18-40	6, 11, 16, 18, 31, 33, 45, 52, 58

Table S1 A summary of the datasets used to fit the HPV transmission model to for pre-vaccination populations.
