# Supplementary material for: Assessing the cost-effectiveness of HPV vaccination strategies for adolescent girls and boys in the UK
Source: BMC Infect Dis. 2019 Jun 24;19:552. doi: 10.1186/s12879-019-4108-y (PMC6591963; doi:10.1186/s12879-019-4108-y)
Supplement: Supplementary file 4 — Table S2. Vaccine uptake in the UK, 2008–2016. (PDF 746 kb) [file 12879_2019_4108_MOESM4_ESM.pdf]

Additional file 4 — Table S2

Vaccine uptake in the UK, 2008-2016.

**Year**	Vaccine	Routine uptake (%)	13	14	15	16	17
2008-09	Bivalent	80.9	0	0	0	0	47.4
2009-10	Bivalent	77.5	0	68.5	68.6	41.7	38.9
2010-11	Bivalent	83.8	4.5	0.3	7.2	2.2	6.4
2011-12	Quadrivalent	87.0	0	0	0	0	0
2012-13	Quadrivalent	85.8	0	0	0	0	0
2013-14	Quadrivalent	88.1	0	0	0	0	0
2014-15	Quadrivalent	87.5	0	0	0	0	0
2015-16	Quadrivalent	85.1	0	0	0	0	0

Table S2 HPV vaccine uptake of girls in the UK over the period 2008-2016, for both the routine campaign (targeting 12-13 year old girls) and the catch-up campaign (targeting those between 13 and 18 years old). Numbers are taken from ([11]) and ([54]), and references therein.
