# Supplementary material for: Assessing the cost-effectiveness of HPV vaccination strategies for adolescent girls and boys in the UK
Source: BMC Infect Dis. 2019 Jun 24;19:552. doi: 10.1186/s12879-019-4108-y (PMC6591963; doi:10.1186/s12879-019-4108-y)
Supplement: Supplementary file 5 — Table S3. Efficacy of the three HPV vaccines against different HPV types. (PDF 48 kb) [file 12879_2019_4108_MOESM5_ESM.pdf]

Additional file 5 — Table S3

Efficacy of the three HPV vaccines against different HPV types.

Vaccine	6	11	16	18	31	33	45	52	58
Bivalent	0	0	100	100	77.1	43.1	79.0	18.9	0.0
Quadrivalent	100	100	100	100	46.2	28.7	7.8	18.4	5.5
Nonavalent	100	100	100	100	100	100	100	100	100

Table S3 Level of protection (%) of the three vaccines against the nine HPV types in the model. Values taken from ([108]).
