# Supplementary material for: Assessing the cost-effectiveness of HPV vaccination strategies for adolescent girls and boys in the UK
Source: BMC Infect Dis. 2019 Jun 24;19:552. doi: 10.1186/s12879-019-4108-y (PMC6591963; doi:10.1186/s12879-019-4108-y)
Supplement: Supplementary file 6 — Table S4. Clinical parameter values and sources. (PDF 50 kb) [file 12879_2019_4108_MOESM6_ESM.pdf]

Additional file 6 — Table S4

Clinical parameter values and sources.

Disease type	% caused by 9 types	6/11	16	18	31/33/45/52/58
Cervical cancer	95.7	0	62.3	18.6	19.1
Anal cancer	84.3	3.6	82.5	5.8	8.1
Vulvar cancer	41.7	4.8	73.5	10.0	11.7
Vaginal cancer	41.7	5.8	69.7	9.9	14.6
Penile cancer	46.9	9.6	68.3	15.4	6.7
Oropharyngeal cancer	31.0	6.7	87.0	2.6	3.8
CIN (grades 2/3)	63.2	0	75.2	9.9	14.8
Genital warts	90	100	0	0	0
RRP	90	100	0	0	0

Table S4 Proportions of the various adverse health effects caused by the nine HPV types. The first column shows percentages of health effects caused by the nine HPV types included in the model; subsequent columns allocate proportions of these percentages to individual types and type groups. All values are taken from reviews by ([57]) and ([22]), except for oropharyngeal cancer ([64]).
