# Supplementary material for: Assessing the cost-effectiveness of HPV vaccination strategies for adolescent girls and boys in the UK
Source: BMC Infect Dis. 2019 Jun 24;19:552. doi: 10.1186/s12879-019-4108-y (PMC6591963; doi:10.1186/s12879-019-4108-y)
Supplement: Supplementary file 7 — Table S5. Costs and health utility decrements. (PDF 55 kb) [file 12879_2019_4108_MOESM7_ESM.pdf]

Additional file 7 — Table S5

Costs and health utility decrements

Parameter	Value	Source
Cost - cervical cancer	£16,527	([69])
Cost - anal cancer	£10,610	([22])
Cost - vulvar/vaginal cancer	£12,446	([22])
Cost - penile cancer	£11,120	([22])
Cost - oropharyngeal cancer	£16,731	([22])
Cost - CIN (grades 2/3)	£385.63	([117])
Cost - genital warts	£281.57	([68])
Cost - RRP	£3,036.01	([67])
Utility decrement (cervical cancer treatment)	0.198	([118])
Utility decrement (anal cancer treatment)	0.355	([118])
Utility decrement (vulvar cancer treatment)	0.223	([118])
Utility decrement (vaginal cancer treatment)	0.223	([118])
Utility decrement (penile cancer treatment)	0.202	([118])
Utility decrement (oropharyngeal cancer treatment)	0.174	([119])
Utility decrement - CIN	0.0261	([120])
Utility decrement - GW episode	0.018	([66])
Utility decrement - RRP episode	1.302	([63])
Permanent utility decrement - cancer recovery	0.0305	([121])
Risk of death - RRP	0.021	([63])

Table S5 Costs and health utility decrements for adverse health effects in the model. Note that costs have been inflated to 2013-14 UK prices.
