# Supplementary material for: Assessing the cost-effectiveness of HPV vaccination strategies for adolescent girls and boys in the UK
Source: BMC Infect Dis. 2019 Jun 24;19:552. doi: 10.1186/s12879-019-4108-y (PMC6591963; doi:10.1186/s12879-019-4108-y)
Supplement: Supplementary file 8 — Figure S1 and Table S6. Parameter distributions. (PDF 176 kb) [file 12879_2019_4108_MOESM8_ESM.pdf]

Additional file 8 — Figure S1 and Table S6

Parameter distributions.

Following the fitting scheme described in the Methods section, the parameter distributions are shown in Fig S1 and Table S6, comparing to literature estimates where possible.

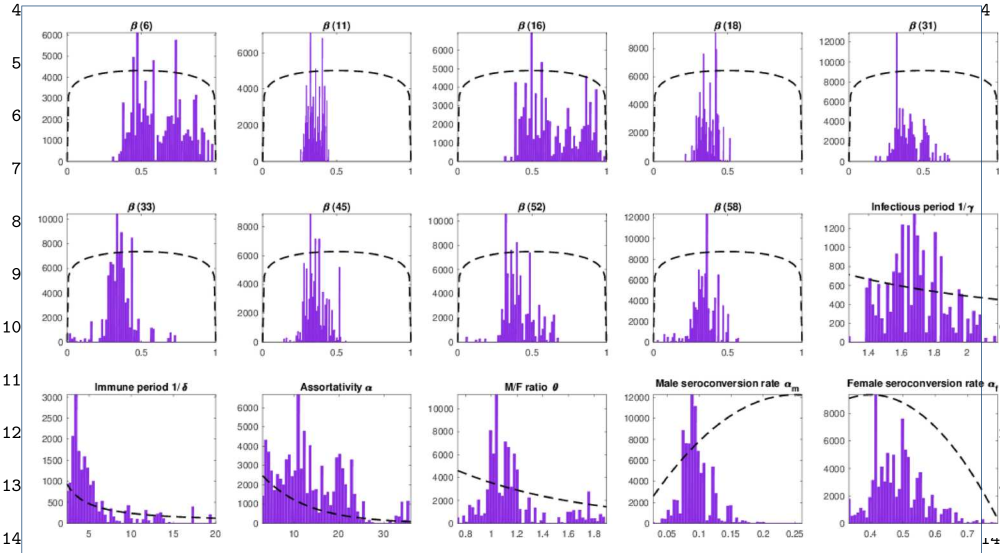


**Figure S1** The parameter distributions for the parameters in the model. Purple histograms are the frequency of parameter values, and black dashed lines are prior distributions. From top to bottom, left to right: plots 1-9 show probabilities $\beta$ of transmitting each type of HPV upon a new partnership being formed; plots 10 and 11 show infectious period $1/\gamma$ and immune period $1/\delta$; plot 12 shows assortativity $\alpha$; plot 13 shows male-to-female ratio of transmission $\theta$; plots 14 and 15 show male seroconversion $\sigma_M$, and female seroconversion $\sigma_F$. Note that plots 10 and 11 are inverted and displayed as time periods rather than rates, for ease of intuition.

Most parameters were well-behaved, and had tight Gaussian-shaped histograms. As expected, types with higher prevalence in the datasets (HPV-6 and 16) had a higher probabilities of transmission upon a partnership being formed than other types. Mean infectious and immune periods were, respectively, approximately 1.7 and 1.4 years. The infectious periods were consistent with those seen in empirical studies ([4]), while there remain questions about the duration (and even existence) of naturally gained immunity ([122], [82]). There was insufficient information in the data to inform the assortativity and male-to-female ratio of transmission, and hence the posteriors for these had less tight histograms. Interestingly, the predicted rates for seroconversion for men and women were lower than the conventional estimates of 30% for men and 65% for women ([96]), with the peaks at roughly 10% for males and 50% for females. This means that the partnership rates taken from Natsal-2 (used for fitting to pre-vaccination prevalences) implied a higher rate of new partnerships, meaning that individuals must have seroconverted at a lower rate to produce the seroprevalence patterns seen in the data. Nevertheless, a higher seroconversion rate was still observed in women.

| Parameter | Value | Literature value | Source |
|---|---|---|---|
| Transmission $\beta_6$ (strain 6) | 0.635 (0.390 - 0.932) | - | - |
| Transmission $\beta_{11}$ (strain 11) | 0.355 (0.281 - 0.437) | - | - |
| Transmission $\beta_{16}$ (strain 16) | 0.647 (0.395 - 0.945) | - | - |
| Transmission $\beta_{18}$ (strain 18) | 0.375 (0.276 - 0.475) | - | - |
| Transmission $\beta_{31}$ (strain 31) | 0.407 (0.279 - 0.619) | - | - |
| Transmission $\beta_{33}$ (strain 33) | 0.362 (0.175 - 0.594) | - | - |
| Transmission $\beta_{45}$ (strain 45) | 0.367 (0.263 - 0.519) | - | - |
| Transmission $\beta_{52}$ (strain 52) | 0.421 (0.297 - 0.648) | - | - |
| Transmission $\beta_{58}$ (strain 58) | 0.352 (0.146 - 0.595) | - | - |
| Infectious period $1/\gamma$ (years) | 1.66 (1.57 - 2.00) | 0.556 - 1.92 | ([103]) |
| Immune period $1/\delta$ (years) | 1.37 (0.296 - 8,86) | Unknown | ([122, 82]) |
| Assortativity $\alpha$ | 13.9 (4.04 - 34.2) | - | - |
| Male:Female ratio $\theta$ | 1.17 (0.854 - 1.80) | 0.593 (0.493 - 0.689 ) | ([51]) |
| Male seroconversion $\sigma_M$ | 9.65% (5.8% - 15%) | 29.3% (27.3% - 31.4%) | ([96]) |
| Female seroconversion $\sigma_F$ | 48.9% (34.7% - 65.4%) | 64.5% (60% - 69%) | ([96]) |

**Table S6** Parameter values with credible intervals, from the distributions in Figure S1. Literature values included where possible.
