# Supplementary material for: Assessing the cost-effectiveness of HPV vaccination strategies for adolescent girls and boys in the UK
Source: BMC Infect Dis. 2019 Jun 24;19:552. doi: 10.1186/s12879-019-4108-y (PMC6591963; doi:10.1186/s12879-019-4108-y)
Supplement: Supplementary file 9 — Figures S2 and S3. Comparing HPV prevalence between the model and data. (PDF 328 kb) [file 12879_2019_4108_MOESM9_ESM.pdf]

Additional file 9 — Figures S2 and S3

Comparing HPV prevalence between the model and data.

Plots showing the trends in HPV prevalence in the model are plotted alongside the various data used in Fig S2 and Fig S3.

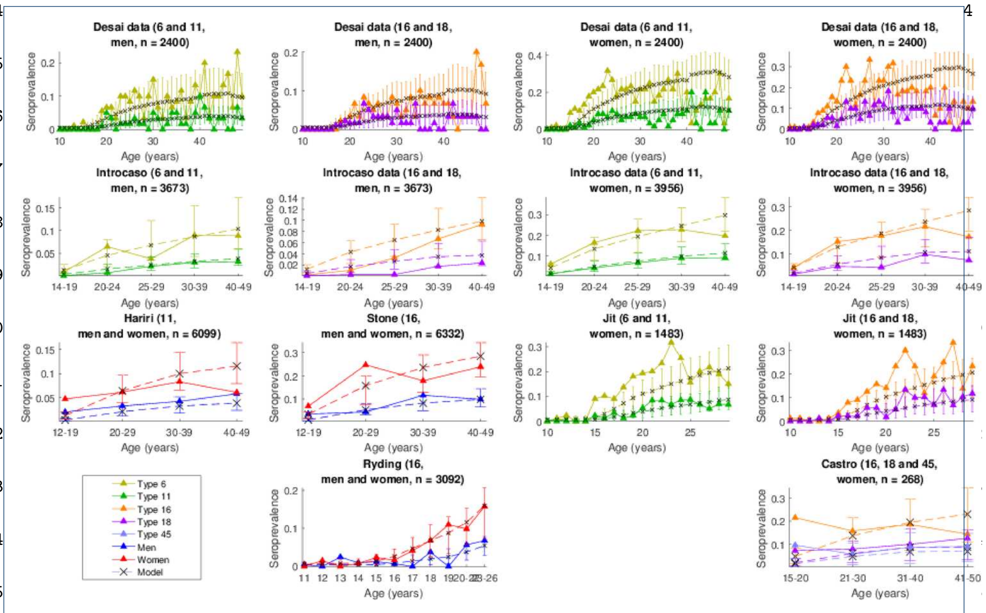

Figure S2 Fitting the epidemiological model to serological data. Seven of the thirteen datasets are serology-based (see Additional file 1 — Appendix S1). Data are shown as solid lines with triangle markers; model means are shown as dashed lines with cross markers, with 95% prediction intervals.

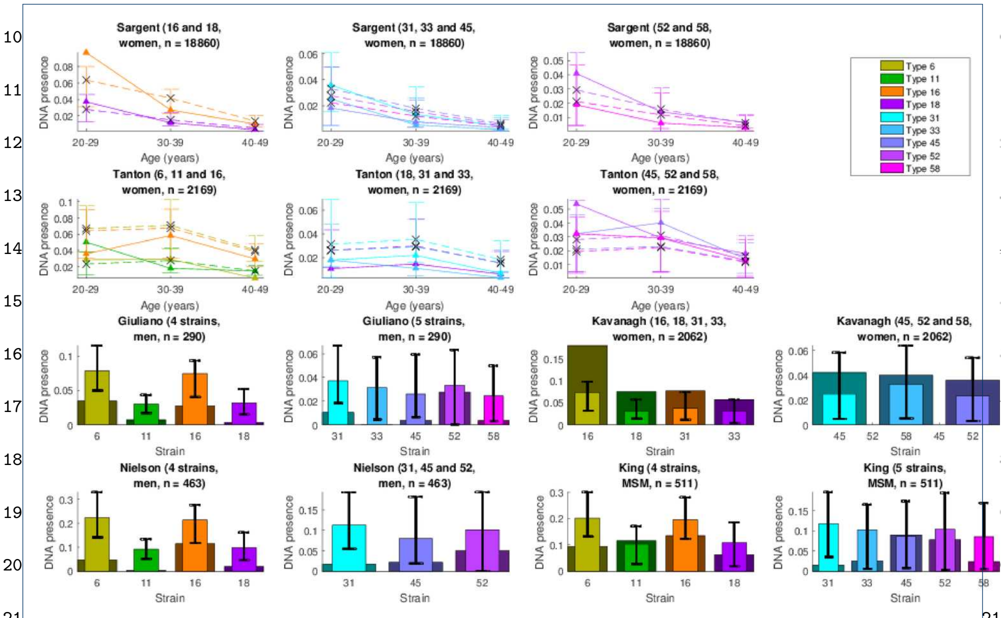

Figure S3 Fitting the epidemiological model to DNA data. Six out of the thirteen datasets are DNA-based (see Additional file 1 — Appendix S1). Data are shown as solid lines with triangle markers, or dark wider bars, as appropriate; model means are shown as dashed lines with cross markers, or lighter narrower bars, with 95% prediction intervals.
