# Supplementary material for: Assessing the cost-effectiveness of HPV vaccination strategies for adolescent girls and boys in the UK
Source: BMC Infect Dis. 2019 Jun 24;19:552. doi: 10.1186/s12879-019-4108-y (PMC6591963; doi:10.1186/s12879-019-4108-y)
Supplement: Supplementary file 10 — Figure S4. The mean threshold price per dose, under different vaccination strategies, for the base case scenario, at both 3.5% and 1.5% discount rate. (PDF 154 kb) [file 12879_2019_4108_MOESM10_ESM.pdf]

Additional file 10 — Figure S4

The mean threshold price per dose, under different vaccination strategies, for the base case scenario, at both 3.5% and 1.5% discount rate.

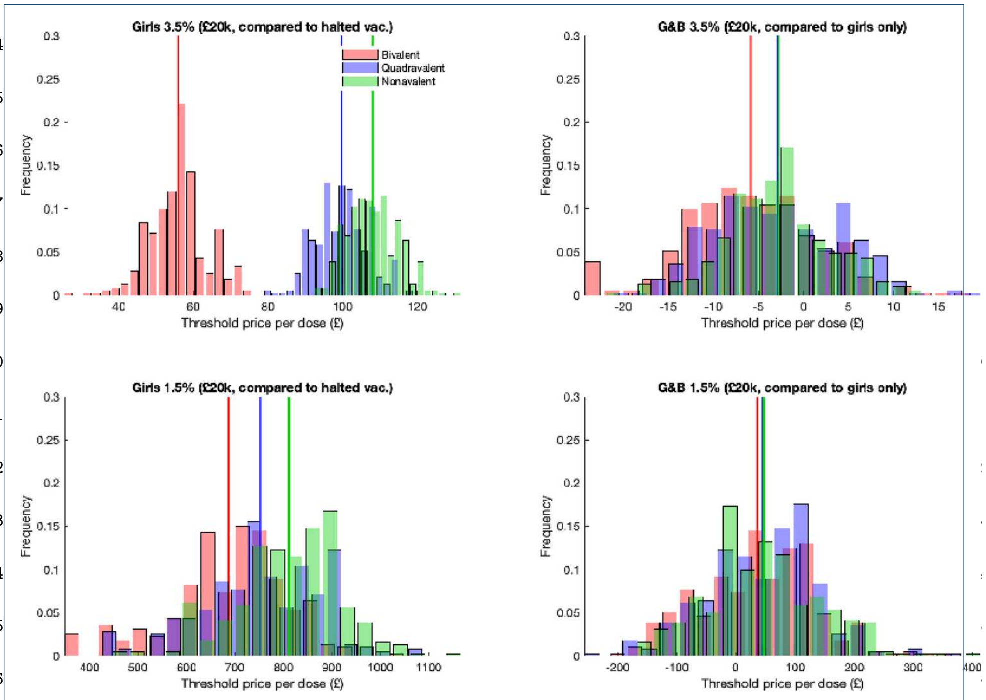

Figure S4 Distribution of threshold dose prices for various vaccination strategies, considering a two-dose schedule. All plots assume £20,000 cost-effectiveness threshold for a QALY, modal parameters from posterior used, and a £10 administration charge per dose. Mean values are shown as vertical lines. Top plots assume 3.5% discount rates applied, bottom plots assume 1.5% discount rates applied. Left plot: comparing girls-only vaccination to halted vaccination. Right plot: comparing gender-neutral vaccination to continuing girls-only vaccination. When comparing a strategy to girls-only vaccination the same vaccine is used for correct comparison.
