# Supplementary material for: Assessing the cost-effectiveness of HPV vaccination strategies for adolescent girls and boys in the UK
Source: BMC Infect Dis. 2019 Jun 24;19:552. doi: 10.1186/s12879-019-4108-y (PMC6591963; doi:10.1186/s12879-019-4108-y)
Supplement: Supplementary file 11 — Table S7. The mean prevalence of different HPV strains (as a percentage) in different ages and genders, after 50 years of simulating a range of vaccination strategies. (PDF 51 kb) [file 12879_2019_4108_MOESM11_ESM.pdf]

Additional file 11 — Table S7

The mean prevalence of different HPV strains (as a percentage) in different ages and genders, after 50 years of simulating a range of vaccination strategies.

Group	Halted vac.	Girls, bi.	Girls, quad.	Girls, nona.	G&B, bi.	G&B, quad.	G&B, nona.
M, 16-25, 16/18	10.735	1.036	1.022	0.999	0.123	0.116	0.113
M, 16-25, 6/11	8.868	8.854	0.676	0.689	8.900	0.091	0.087
M, 16-25, 31/33/45/52/58	15.132	8.858	11.949	1.046	7.037	9.323	0.156
M, 26-35, 16/18	5.866	1.200	1.223	1.187	0.436	0.417	0.423
M, 26-35, 6/11	5.148	5.234	0.984	0.955	5.272	0.366	0.341
M, 26-35, 31/33/45/52/58	10.183	6.575	8.588	2.046	5.222	7.214	0.658
M, 36-50, 16/18	3.102	1.275	1.269	1.251	1.066	1.010	1.050
M, 36-50, 6/11	3.038	3.078	1.371	1.354	3.108	1.256	1.214
M, 36-50, 31/33/45/52/58	5.988	4.621	5.456	2.667	4.153	5.041	2.202
F, 16-25, 16/18	7.992	0.328	0.317	0.303	0.113	0.111	0.115
F, 16-25, 6/11	6.678	6.665	0.160	0.162	6.738	0.091	0.088
F, 16-25, 31/33/45/52/58	11.651	6.299	8.694	0.313	5.470	7.287	0.160
F, 26-35, 16/18	3.907	0.356	0.366	0.336	0.150	0.142	0.143
F, 26-35, 6/11	3.368	3.372	0.239	0.222	3.399	0.144	0.134
F, 26-35, 31/33/45/52/58	6.407	3.721	5.160	0.535	3.243	4.490	0.342
F, 36-50, 16/18	1.780	0.693	0.693	0.681	0.536	0.544	0.543
F, 36-50, 6/11	1.868	1.854	0.849	0.854	1.871	0.811	0.808
F, 36-50, 31/33/45/52/58	3.411	2.682	3.129	1.556	2.493	2.891	1.479

Table S7 The average prevalence of HPV (as a percentage of the population), by gender (M/F), age (16-25, 26-35 and 36-50) and strain (16/18, 6/11 and 31/33/45/52/58), over 200 simulations. For each simulation numbers are taken 50 years after the vaccination strategy has been in place. The strategies considered are: halted vaccination, and for vaccinating either girls or girls and boys together, using one of the three vaccines (bi. = bivalent, quad. = quadrivalent, nona. = nonavalent).
