# Supplementary material for: Assessing the cost-effectiveness of HPV vaccination strategies for adolescent girls and boys in the UK
Source: BMC Infect Dis. 2019 Jun 24;19:552. doi: 10.1186/s12879-019-4108-y (PMC6591963; doi:10.1186/s12879-019-4108-y)
Supplement: Supplementary file 12 — Table S8. Cases of disease for different vaccination strategies. (PDF 1254 kb) [file 12879_2019_4108_MOESM12_ESM.pdf]

Additional file 12 — Table S8

Cases of disease for different vaccination strategies.

**Health effect**	Halted vac.	Girls, bi.	Girls, quad.	Girls, nona.	G&B, bi.	G&B, quad.	G&B, nona.
Cervical cancer	8.259	1.627	1.801	0.767	1.310	1.460	0.495
Anal cancer	3.354	1.200	1.196	1.025	1.124	1.011	0.838
Vulvar cancer	3.291	2.459	2.291	2.143	2.304	2.346	2.249
Vaginal cancer	0.641	0.489	0.491	0.409	0.462	0.466	0.434
Penile cancer	1.946	1.416	1.393	1.319	1.405	1.167	1.315
Oropharyngeal cancer	24.264	19.214	18.877	18.698	18.524	18.441	17.857
CIN (grades 2/3)	80.720	35.356	36.450	30.588	34.307	34.965	29.893
Genital warts	243.837	244.175	64.457	64.271	243.268	53.931	53.110
RRP	0.091	0.089	0.023	0.017	0.060	0.030	0.019

Table S8 The average number of cases of different health effects per 100,000 individuals, over 500 simulations. For each simulation annual numbers are averaged over 51 and 70 years after the vaccination strategy has been in place. The strategies considered are: halted vaccination, and for vaccinating either girls or girls and boys together, using one of the three vaccines (bi. = bivalent, quad. = quadrivalent, nona. = nonavalent).
