# Supplementary material for: Assessing the cost-effectiveness of HPV vaccination strategies for adolescent girls and boys in the UK
Source: BMC Infect Dis. 2019 Jun 24;19:552. doi: 10.1186/s12879-019-4108-y (PMC6591963; doi:10.1186/s12879-019-4108-y)
Supplement: Supplementary file 13 — Tables S9 and S10, and Figure S5. Incremental cost-effectiveness ratios. (PDF 146 kb) [file 12879_2019_4108_MOESM13_ESM.pdf]

Additional file 13 — Tables S9 and S10, and Figure S5

Incremental cost-effectiveness ratios.

An alternative to calculating the threshold dose price is to set the vaccine dose price to a fixed value, and then calculate the incremental cost of each additional QALY for a given vaccination strategy (the incremental cost-effectiveness ratio). These are presented for a range of dose prices (covering the list price for the quadrivalent vaccine of £87.50 per dose), using each of the three vaccines, and vaccinating either girls only or girls and boys. The results are shown in Tables S9 and S10, and Fig S5.

**Strategy**	£0	£20	£40	£60	£80	£100
Girls, bivalent	strategy dominant	strategy dominant	1943	15930	29916	43903
Girls, quadrivalent	strategy dominant	strategy dominant	strategy dominant	1364	8877	16390
Girls, nonavalent	strategy dominant	strategy dominant	strategy dominant	strategy dominant	6655	13668
Girls & boys, bivalent	strategy dominant	109	26499	52890	79280	105671
Girls & boys, quadrivalent	strategy dominant	strategy dominant	7007	21152	35297	49442
Girls & boys, nonavalent	strategy dominant	strategy dominant	4997	18195	31394	44592

Table S9 Incremental cost-effectiveness ratios for alternative vaccination strategies at alternative assumed vaccine dose prices (minus the £10 administration fee assumed in the analysis - hence the first column represents paying the administration fee plus zero for each vaccine dose). Evaluated strategies are relative to halted vaccination. Note that "strategy dominant" means that the vaccination strategy is on average both cheaper and more effective.

**Strategy**	£0	£20	£40	£60	£80	£100
Girls & boys, bivalent	strategy dominant	204344	439225	674106	908987	1143868
Girls & boys, quadrivalent	strategy dominant	98425	219913	341401	462889	584377
Girls & boys, nonavalent	strategy dominant	91142	203735	316327	428920	541513

Table S10 Incremental cost-effectiveness ratios for alternative vaccination strategies at alternative assumed vaccine dose prices (minus the £10 administration fee assumed in the analysis). Evaluated strategies are relative to vaccinating girls only.

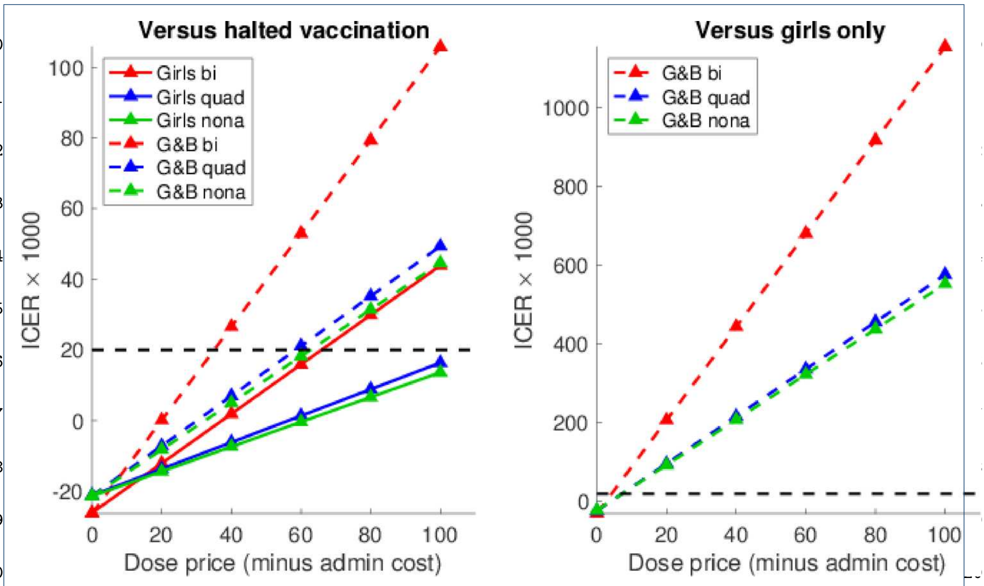

Figure S5 The incremental cost-effectiveness ratios, for all three vaccines, at a range of dose prices. Values for girls-only vaccination are shown by solid lines, gender-neutral by dashed lines. Vaccines are colour coded: red (bivalent), blue (quadrivalent), green (nonavalent). £20,000 threshold shown by horizontal dashed black line. Left plot: compared to halted vaccination. Right plot: compared to girls-only vaccination.
